# An extremely rare case of a gastric accessory spleen: case report and review of the literature

**DOI:** 10.1186/s12876-021-01852-z

**Published:** 2021-07-06

**Authors:** Guiqin Chen, Lei Nie, Tijiang Zhang

**Affiliations:** Department of Radiology, Medical Imaging Center of Guizhou Province, The Affiliated Hospital of Zunyi Medical University, Zunyi, 563000 China

**Keywords:** Accessory spleen, Gastric, Gastroscopy and endoscopic ultrasonography, Computed tomography, Gastric stromal tumor

## Abstract

**Background:**

The accessory spleen has no anatomical or vascular relationship with the normal spleen, The tissue structure and physiological function of the accessory spleen are the same as those of the normal spleen, which usually locate in the splenic hilum and the tail of the pancreas. The aims of this manuscript are to present a rare case of the gastric accessory spleen and a review of the literature.

**Case presentation:**

A 19-year-old male patient was sent to the emergency department with stomach bleeding after drinking alcohol. The computed tomographic scan showed a 1.2 cm × 1.7 cm mass at the lesser curvature of the gastric fundus. Gastrointestinal endoscopy displayed a submucosal elevated lesion on the gastric fundus, and gastrectomy was performed. Postoperative pathological examination proved an accessory spleen in the stomach. The postoperative course was uneventful, and the patient was discharged on the 6th day after the surgery.

**Conclusions:**

The accessory spleen at the fundus of stomach is extremely rare, especially in this case, which is accompanied by acute gastric bleeding, and it is difficult to diagnosis before operation. Many literatures reported that it was misdiagnosis as tumor, so it is necessary to diagnose accessory spleen correctly.

## Background

The spleen is derived from mesenchymal cells of the dorsal mesentery,and appears approximately at the sixth week of embryologic life [1, 2]. The spleen is located between the 9th and 11th left ribs in abdominal cavity, between the gastric bottom and the left diaphragm. With a weight of approximately 200 g, it represents the largest lymphoid organ in the body [3]. At the fifth week of embryonic development, an accessory spleen may form if the embryo spleen bud is not fully fused or its single cell is separated from the body of the spleen, which is a congenital defect [2, 4]. Accessory spleen is common and typical in most imaging manifestations. However, the imaging manifestation of accessory spleen in this case is not typical, and it is difficult to distinguish it from gastric fundus tumor. The purposes of this manuscript are to present a case of a patient with a rare gastric accessory spleen and to update the literature concerning this rare disease.

## Case presentation

**Fig. 1:**
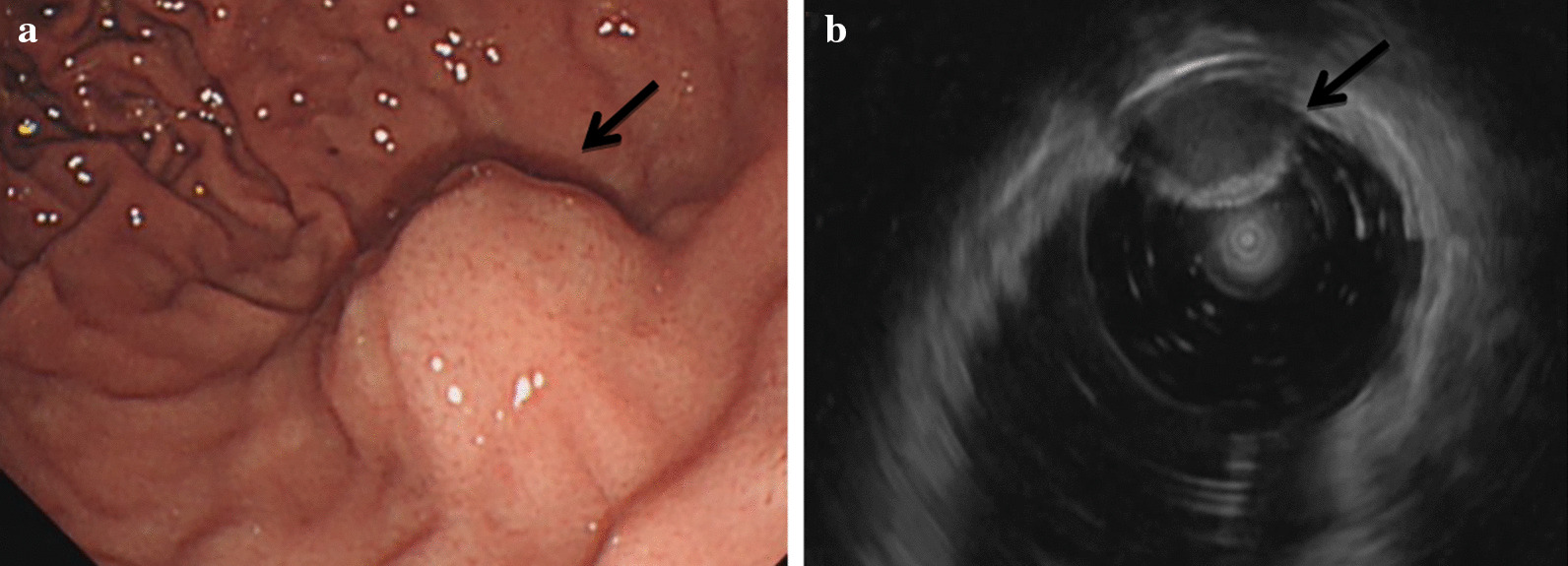
A 19-year-old male patient with stomach bleeding after drinking alcohol in 2020. Gastroscopy revealed a submucosal elevated lesion in the gastric fundus. Endoscopic ultrasonography revealed a tumor with low homogenous echogenicity originating in the gastric muscular layer. **a** Gastroscopy; **b** endoscopic ultrasonography

**Fig. 2:**
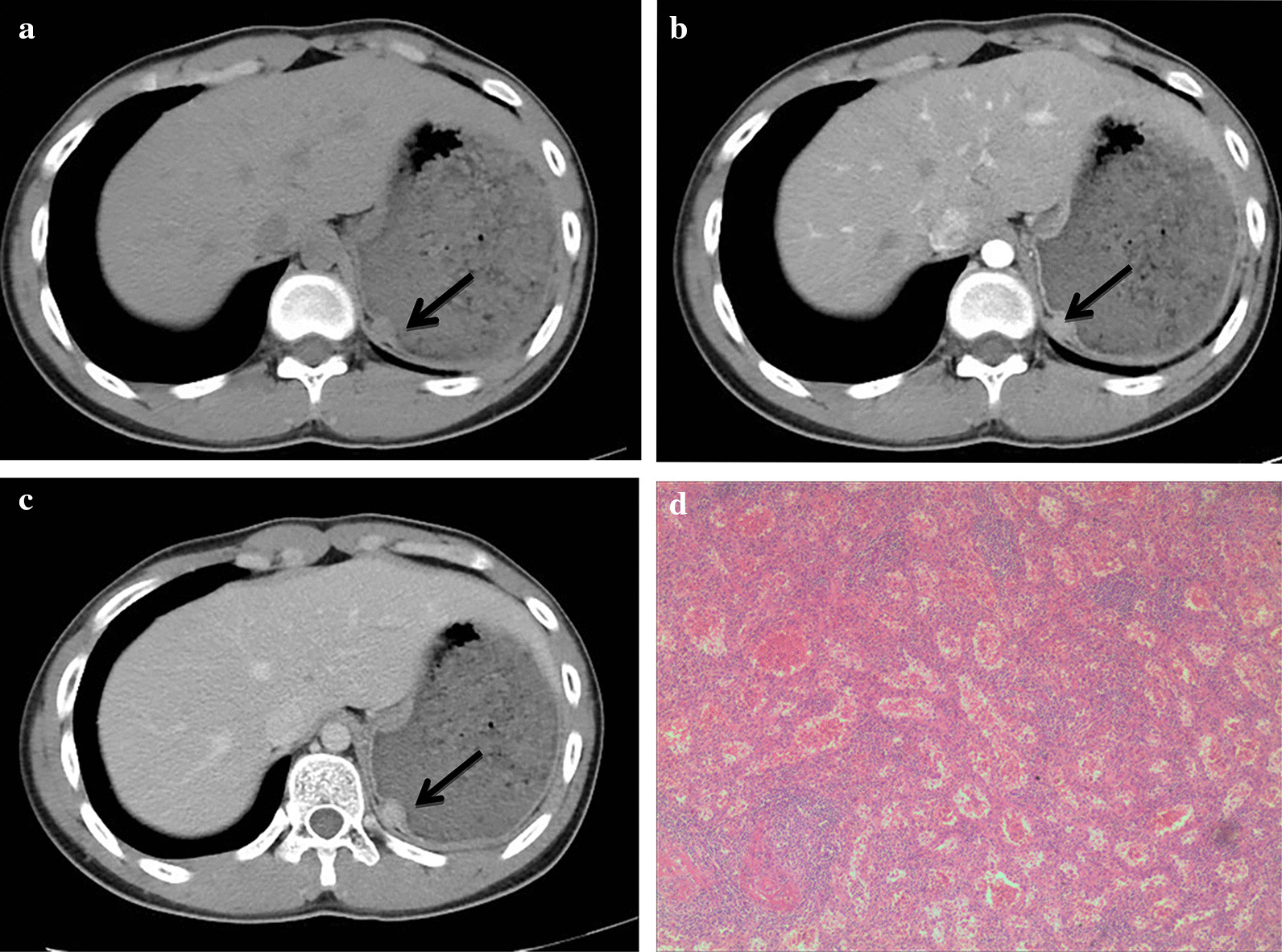
Routine CT examination found a small well-marginated ovoid nodule in the gastric fundus,the nodule showed obvious homogeneous enhancement on the dual-phase enhanced CT scan. Histopathology showed the tissue was mainly composed of lymphocyte nodules and surrounded with sinusoids containing red blood cells and histological structure was consistent with that of an accessory spleen. **a** Axial unenhanced CT image; **b** Axial enhanced CT image in the arterial stage; **c** axial enhanced CT image in the portal vein stage; **d** Hematoxylin and eosin staining (× 200)

In November 2020, a 19-year-old man was sent to the emergency department for stomach bleeding after drinking alcohol. There was no hematemesis, melena, hematuria, hematochezia, dizziness and headache. Physical examination showed that there was no obvious gastrointestinal type and peristalsis wave, no abdominal tenderness and rebound pain. The patient was hemodynamic stability (blood pressure 120/74mmHg, pulse rate 75/min). therefore, laboratory tests, gastroscopy and Computed tomography (CT) scan were performed. Gastroscopy showed a submucosal elevated lesion in the gastric fundus (Fig. 1a). Endoscopic ultrasonography revealed a tumor with low homogenous echogenicity originating in the gastric muscular layer (Fig. 1b), considering the posibility of stromal tumors. CT scan of the abdomen demonstrated a high density lesion measuring 1.2 cm × 1.7 cm in the gastric fundus, the nodule showed obvious homogeneous enhancement on the dual-phase enhanced CT scan, and stromal tumors or neurogenic tumors were considered (Fig. 2a–c). No abnormality was showed on laboratory examinations. We planned to perform a laparoscopic resection of the gastric fundus tumor, with the patient in the supine position and under general anesthesia, the transverse supraumbilical incision was made, a laparoscope was placed through a supraumbilical incision. An ultrasonic knife was also used to cut the gastrocolic ligament along the greater curvature of the stomach, cut and ligate the root of the left gastroepiploic artery and vein, and individually cut the first short gastric vessels, to expose the fundus of stomach. We found the tumor protrudes from the anterior wall of the fundus, on the lesser curvature of the stomach, measuring 2.0  × 2.0 cm in diameter, with a clear boundary and protruding serosa. The tumor was lifted by non-invasive forceps, closed and cut along the edge of the tumor at about 1 cm, the tumor was removed completely, and the edge of the gastric wall was strengthened by continuous suture. The abdominal cavity was rinsed with saline and drained, with the drainage tube in the left upper abdomen, and there was no active bleeding. Postoperative pathological examination proved an accessory spleen in the stomach (Fig. 2d), the size of the nodule is about 1.6 cm × 1.1 cm × 0.8 cm. After postoperative anti-inflammatory, hemostatic and other symptomatic support treatment, and the patient recovered well and the patient was discharged on the 6th day after surgery. He was followed up by telephone for two months, during which he had no complications.

## Discussion and conclusions

**Fig. 3:**
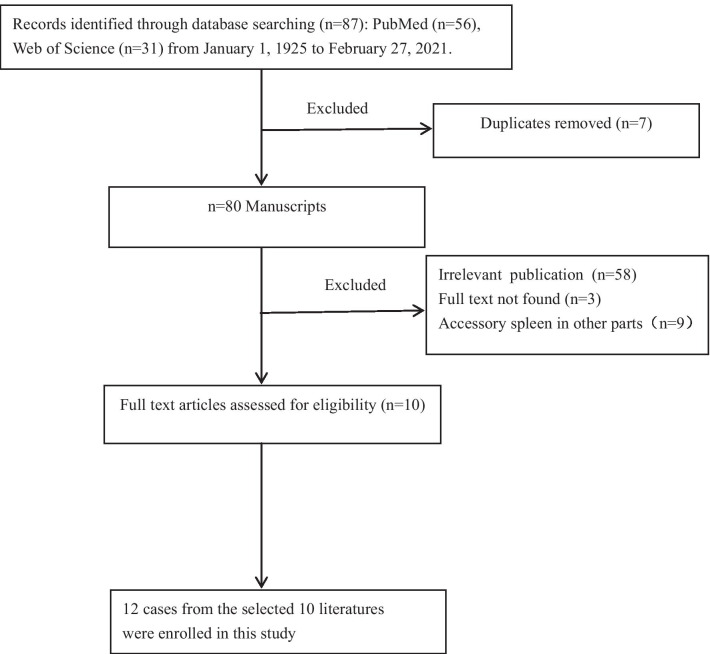
The flow chart of the literature screening process for gastric accessory

To perform the review of the literature, relevant articles in English were extensively searched from the PubMed, Web of Science database. The period of research was between 1921 and 2021. The date of the last search was February 27, 2021. The keywords used for the search were“stomach”, “stomachs”, “gastric”, “accessory spleen”, “splenculus” “splenulus”. These words were used individually “OR” with the Boolean operator “AND”. A total of 87 articles were analyzed from 1921 to 2021. The flow chart of the literature screening process is set out in Fig. 3. A total of 10 articles involving 12 cases were included for analysis. For each case, the data were collected for the first author, year of publication, patient’s age, sex, size, symptoms, location, imaging characteristics, and follow-up results (Table 1).

**Table 1:** The case of gastric accessory spleen from the literature review

Case nos.	Author/ year	Age	Sex	Presentation	Location	Size (cm)	CT	Follow-up (year)
1	Tkdasgupta/1960	33	F	Epigastric pain	Gastric fundus	–	–	–
2	ShunzenChin/ 2007	62	M	Abdominal discomfort	Gastric fundus	6(CT)	Well-marginated ovoid mass	–
3	Ji-YongAhn/ 2012	39	F	–	Gastric fundus	1.9(CT)	A well-marginated and enhanced ovoid mass	0.6
4	Guang-yaoWang/2014	61	M	Abdominal discomfort	Gastric fundus	2.6 × 1.9(CT)	A well-marginated ovoid mass	–
5	Weijie-Wang/ 2015	40	M	Abdominal discomfort	Gastric fundus	–	A well-marginated ovoid mass	–
6	Weijie-Wang/ 2017	40	M	Abdominal discomfort	Gastric fundus	–		–
7	Sulaiman-Almazeedi/ 2017	54	F	–	Gastric fundus	2.5 × 2.0 × 0.7 (CT)	–	–
8	Jin SukKim/ 2018	52	M	–	Gastric fundus	2.4(CT)	A well-marginated and homogeneous enhanced ovoid mass	3
9	Ya Ting-Shen/ 2018	34	F	Epigastric discomfort	Gastric fundus		An oval mass with homogenous	–
10	Ya TingShen/ 2018	65	F	Epigastric pain	Gastric body	–	An oval mass with homogenous	–
11	Jing Zhang/2020	34	M	Abdominal pain.	Gastric fundus	1.8 × 1.2(US)	–	–
12	Hugo J R Bonatti/2020	66	M	Abdominal discomfort	Gastric fundus	–	–	–

F, female; M, male; His, history; CT, computed tomography; US, ultrasound

According to the literature, accessory spleen are most commonly found in the splenic hilum and the tail of the pancreas and are rare in the stomach. The 12 reported cases of gastric accessory spleen including 5 females and 7 males, with a female to male ratio of 0.71 to 1. the age distribution of the patients was mainly between 30 and 70 years old, and these patients predominantly suffered from abdominal discomfort, with lesions mostly located in the gastric fundus. Most accessory spleens usually appears as a well-bundary ovoid mass, 1–3 cm in diameter, masses larger than 4 cm are very rare.

In this case report, we reported a case of a 19-year-old man who developed a nodule in the gastric fundus. Due to the lesion is closely related to the fundus of stomach, clinicians were highly suspected that it was a gastric fundus stromal tumor. As mentioned in the literature, gastric stromal tumor is the most common mesenchymal tumors of the digestive tract [5, 6], The diagnosis of gastric stromal tumor was supported by the imaging features and the gastroscopy. Consequently, we provided a diagnosis of Gastric stromal tumor. However, the pathological result after the operation was an accessory spleen.

An accessory spleen is defined as ectopic splenic tissue that due to the failure of cell fusion during embryonic development while migrating from the midline to the left upper quadrant [7, 8]. An accessory spleen includes isolated spleen tissue outside the normal spleen [9, 10], usually located in the splenic hilum and the tail of the pancreas, but occasionally in the greater omentum and gastrointestinal tract [11]. An ectopic accessory spleen in the stomach is relatively rare which is usually asymptomatic and incidentally discovered. This is the case of an accessory spleen that was found incidentally on the fundus of the stomach due to gastric bleeding. The arterial system for the fundus varies and is made up of direct or indirect vascular supply which may arise from 9 arteries: the left inferior diaphragmatic, accessory left hepatic, left gastric, left adrenal, splenic, posterior gastric, superior polar, left gastro-epiploic and the gastro-splenic arteries [12]. Imaging examinations, such as CT, and magnetic resonance imaging (MRI), play an important role in the diagnosis of accessory spleen. CT scans show a well-margined single and multiple nodules,which is similar to that of normal splenic parenchyma, contrast-enhanced dynamic CT of the spleen shows a normal mottled or heterogeneous enhancement pattern during the arterial phase (AP) and the same degree of enhancement was found in portal vein phase, delayed phase. MRI can also be used to evaluate tissue aspects and the vascular pedicle of the accessory spleen. Nuclear medicine imaging can confirm the diagnosis with scintigraphy performed with 99mTC-labelled colloids or TC-99 m heat damaged red blood cells because the colloid labelled with TC-99 m is taken from the reticulum-endothelium and makes visible the spleen, liver and bone marrow [1]. The spleen has a high uptake rate of 99mTC, which is characterized by radioactive accumulation. Enhanced CT scan and nuclear medicine may be helpful for distinguishing between gastrointestinal tumors and accessory spleen.

At present, there are many clinical cases of accessory spleen misdiagnosed as tumor [9]. This lesion is located in the lesser curvature of the gastric fundus, which presses the gastric wall and the boundary between the tumor and adjacent gastric wall of the lesser curvature is unclear, the differential diagnoses of gastric stromal tumors, lymphomas, and metastatic tumor are still challenging. Gastric stromal tumors: CT examination identifies soft tissue mass with uniform density at the distal stomach, and most of the benign tumors are less than 5 cm, clearly defined from the surrounding structure, and could grow inside and outside the gastric cavity. We reported a case that accessary spleen had shown the presence of a gut mass that resembled a gastrointestinal stromal tumor on CT scan. Most gastric lymphomas tend to be fused, and the enhancement degree of CT is weak. The imaging manifestations of metastatic tumor and gastric accessory spleen are different, which have primary lesions, but the focus is usually asymptomatic when the focus is small. And in these cases, it is difficult to distinguish accessory spleen from gastric tumor. Therefore, despite its rarity, accessory spleen should be necessarily considered in the differential diagnosis when a gastric fundus nodule is incidentally found in a patient.

Finally, if the accessory spleen is small and the patient has not any symptoms, there is no need for surgery. Surgical treatment should be performed if showing symptoms due to greater compression or torsion. All of the patients reported with gastric accessory spleen underwent surgery. In recent years, laparoscopic space-occupying lesions in the stomach have been primarily considered over open surgery because laparoscopic surgery is less invasive, has fewer complications and a lower mortality rate than open surgery.

The position of accessory spleen may be different and gastric accessory spleen is a rare entity that arises as a result of a birth defect or an acquired condition. Preoperative diagnosis of accessory spleen is still difficult due to a lack of specificity in imaging studies which is similar to the imaging findings of some tumors in the stomach, Especially in an emergency, as our case, the accessory spleen may be removed by mistake. Although gastric accessory spleen is uncommon, it should be considered as one of the differential diagnoses of gastric diseases. While postoperative histopathologic examination is still the gold standard, fine nuclear medicine imaging and enhanced CT scan may be useful in the diagnosis of accessory spleen.

## Data Availability

All patients’ data and medical images can be found in the database of Information Office of Affiliated Hospital of Zunyi Medical University.

## Abbreviations

CT: Computed tomography
MRI: Magnetic resonance
AP: Arterial phase
